# Chemoradiation Increases PD-L1 Expression in Certain Melanoma and Glioblastoma Cells

**DOI:** 10.3389/fimmu.2016.00610

**Published:** 2016-12-22

**Authors:** Anja Derer, Martina Spiljar, Monika Bäumler, Markus Hecht, Rainer Fietkau, Benjamin Frey, Udo S. Gaipl

**Affiliations:** ^1^Department of Radiation Oncology, Universitätsklinikum Erlangen, Friedrich-Alexander-Universität Erlangen-Nürnberg, Erlangen, Germany; ^2^Department of Cell Physiology and Metabolism, Faculty of Medicine, Centre Medical Universitaire (CMU), University of Geneva, Geneva, Switzerland

**Keywords:** fractionated radiotherapy, immunotherapy, checkpoint inhibitor, PD-L1, IFN-gamma, IL-6, melanoma, glioblastoma

## Abstract

Immunotherapy approaches currently make their way into the clinics to improve the outcome of standard radiochemotherapy (RCT). The programed cell death receptor ligand 1 (PD-L1) is one possible target that, upon blockade, allows T cell-dependent antitumor immune responses to be executed. To date, it is unclear which RCT protocol and which fractionation scheme leads to increased PD-L1 expression and thereby renders blockade of this immune suppressive pathway reasonable. We therefore investigated the impact of radiotherapy (RT), chemotherapy (CT), and RCT on PD-L1 surface expression on tumor cells of tumor entities with differing somatic mutation prevalence. Murine melanoma (B16-F10), glioblastoma (GL261-luc2), and colorectal (CT26) tumor cells were treated with dacarbazine, temozolomide, and a combination of irinotecan, oxaliplatin, and fluorouracil, respectively. Additionally, they were irradiated with a single dose [10 Gray (Gy)] or hypo-fractionated (2 × 5 Gy), respectively, norm-fractionated (5 × 2 Gy) radiation protocols were used. PD-L1 surface and intracellular interferon (IFN)-gamma expression was measured by flow cytometry, and IL-6 release was determined by ELISA. Furthermore, tumor cell death was monitored by AnnexinV-FITC/7-AAD staining. For first *in vivo* analyses, the B16-F10 mouse melanoma model was chosen. In B16-F10 and GL261-luc2 cells, particularly norm-fractionated and hypo-fractionated radiation led to a significant increase of surface PD-L1, which could not be observed in CT26 cells. Furthermore, PD-L1 expression is more pronounced on vital tumor cells and goes along with increased levels of IFN-gamma in the tumor cells. In melanoma cells CT was the main trigger for IL-6 release, while in glioblastoma cells it was norm-fractionated RT. *In vivo*, fractionated RT only in combination with dacarbazine induced PD-L1 expression on melanoma cells. Our results suggest a tumor cell-mediated upregulation of PD-L1 expression following in particular chemoradiation that is not only dependent on the somatic mutation prevalence of the tumor entity.

## Introduction

A promising new cancer treatment strategy is combining classical radiochemotherapy (RCT, chemoradiation) with immunotherapy (IT). Even it is known since long time that RCT does not induce complete immune suppression and that besides temporarily restricted leukopenia and granulocytopenia, the remaining immune cells preserve their function (1), only in the recent years preclinical and clinical research focused on combination of RCT with IT (2). As certain chemotherapeutic agents such as anthracyclines (3), also ionizing radiation is capable of rendering the tumor cell and its microenvironment immunogenic by inducing the upregulation of activation markers for immune cells and death receptors on tumor cells and by further inducing the release of danger signals and cytokines (4–6).

However, besides these immune-stimulating properties of radiation, it can also induce the upregulation of immune suppressive molecules. The programed cell death receptor ligand 1 (PD-L1, CD274, or B7-H1) is one prominent example for this. Under normal physiological conditions, PD-L1 is constitutively expressed on immune cells, including dendritic cells (DCs), as well as on non-hematopoietic cells (7) and helps to maintain self-tolerance. Upon binding to its inhibitory receptor, programed death receptor 1 (PD-1) (8), T cells are impaired (9). Many tumor entities show a constant PD-L1 surface expression and thereby evade immune surveillance (7, 10). The pro-inflammatory cytokine interferon (IFN)-gamma has been shown to induce upregulation of PD-L1 on the surface of tumor cells (11).

Therefore, blocking either the immune checkpoint protein PD-1 or its ligands PD-L1 and/or PD-L2 are new anticancer treatment strategies that have already been shown to be successful (12). Durable responses occurred in 30–35% of patients with advanced melanoma (13–15), and consecutively many clinical and preclinical studies for other tumor entities such as lung (16), breast (17, 18), and bladder (19) were initiated.

In particular, to exploit the radiation-induced increased endogenous antitumor immune responses, the increased expression of PD-L1 on tumor cells or infiltrating immune cells has to be counteracted by blocking the PD-1/PD-L1 pathway (20). For this, knowledge about expression of PD-L1 on tumor cells after in particular RCT is mandatory to adapt multimodal therapies for the most beneficial induction of antitumor immunity.

Of note is that targeting PD-L1 is not equally successful in every patient and should have PD-L1 surface expression on the tumor cells as prerequisite (21). Studies examining PD-L1 expression in murine tumor models have already shown that radiation can induce an upregulation of PD-L1 on tumor cells as unwanted side effect. It is mostly mediated by IFN-gamma-producing T cells (22). Furthermore, chemoradiation led to increased PD-1 expression on CD4+ T cells in the peripheral blood of patients with human papillomavirus-related oropharyngeal cancer (23).

However, only little is known which RCT protocol induces immunogenic tumor cell death and further leads to increased PD-L1 expression on tumor cells of a distinct tumor entity and thereby renders blockade of the PD-1/PD-L1 pathway reasonable. We therefore investigated the effect of tumor entity-related RCT schemes on the induction of cell death and concomitant PD-L1 expression on viable and apoptotic tumor cells. The latter are immune suppressive since they expose phosphatidylserine (24). This could be further enhanced by additional expression of PD-L1, which is again counterproductive for antitumor immune responses.

The somatic mutation prevalence of tumor cells is highly connected with the tumor cells immunogenicity. Mutations might lead to the generation of neoantigens against which an immune response is started (25). Radiotherapy (RT) further contributes to the generation of neoantigens, and neoantigen-specific CD8+ T cell responses have been shown to go along with tumor regression (26). Since melanoma has the highest somatic mutation prevalence it does respond very well to IT. We therefore focused in our preclinical examinations on this tumor entity and compared it with one displaying only intermediate somatic mutation prevalence, namely colorectal cancer (27). Additionally, glioblastoma cells were included to get hints about RCT-induced modulation of the immunological tumor cell phenotype of a tumor entity located at an immune-privileged organ, namely the brain.

## Materials and Methods

### Cell Culture and Reagents

Established murine melanoma (B16-F10, ATCC, USA), glioblastoma (GL261-luc2, Caliper, USA), and colorectal carcinoma (CT26, ATCC, USA) cell lines were used. B16-F10 and CT26 cells were maintained in RPMI 1640 medium (Sigma, USA), supplemented with 10% heat-inactivated FCS, 100 U/ml penicillin, and 100 µg/ml streptomycin (Gibco, USA). GL261-luc2 cells were cultured in high glucose Dulbecco’s Modified Eagle’s Medium (Gibco, USA) supplemented with heat-inactivated 10% FCS (Biochrom, Germany) and 0.5% geneticin (Gibco, USA). Cells were grown in cell culture flasks (Greiner BioOne, Germany) in a humidified chamber at 37°C and 5% CO_2_. All cell lines were tested to be free of mycoplasma contamination. Irinotecan, oxaliplatin, and fluorouracil were purchased as ready-to-use infusions. Temozolomide (TMZ, Sigma-Aldrich, USA) was dissolved at a stock concentration of 100mM in dimethylsulfoxide (Roth, Germany) and stored at −20°C. Dacarbazine (DTIC, Sigma-Aldrich, USA) was dissolved in culture medium before use. Chemotherapeutics were diluted in the respective medium before cell treatment. As a positive control for PD-L1 induction (0.5 ng/ml), recombinant murine interferon-gamma (rmIFN-γ, R&D Systems, USA) was administered to otherwise non-treated cell cultures.

### Treatment of Tumor Cell Lines

Cells were seeded at a density of 20,000–25,000 B16-F10 cells, 30,000 CT26 cells, and 100,000 GL261-luc2 cells per 25 cm^2^. After resting overnight, tumor cells were subjected to chemotherapeutic treatments and radiation. In brief, B16-F10 cells were treated with a single dose of 250µM DTIC on day 1, GL261-luc2 cells were treated with 20µM TMZ every other day for 5 days, and CT26 cells were incubated with single doses of 10 µg/ml irinotecan, 10 µg/ml oxaliplatin, and 400 ng/ml 5-fluorouracil for 4 h, before chemotherapy (CT) was washed off using Dulbecco’s phosphate-buffered saline (Gibco, Germany) and cells were cultured in fresh medium.

**Figure 1:**
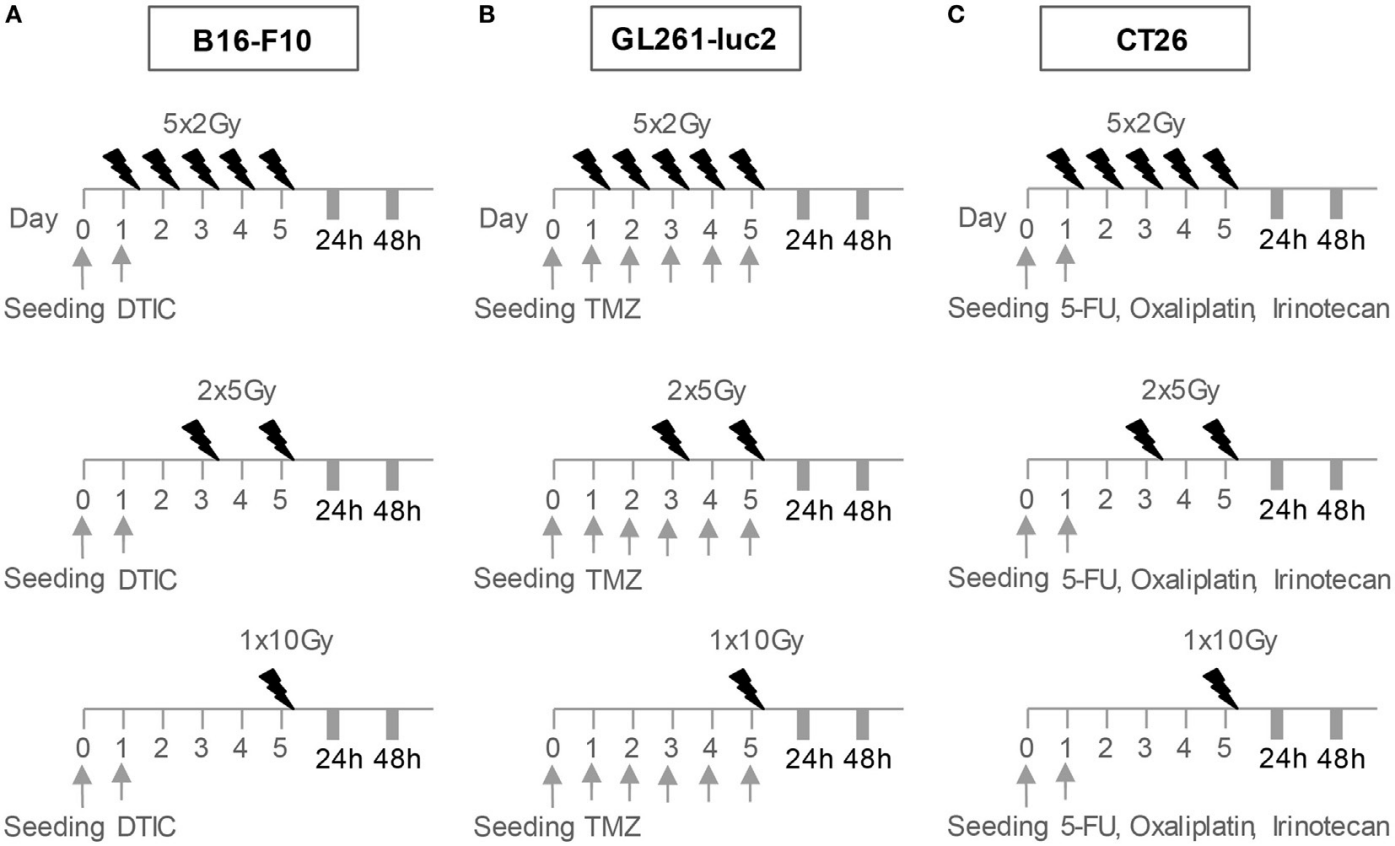
**Radiation and chemotherapy (CT) treatment scheme for the cell lines B16-F10 (A), GL261-luc2 (B), and CT26 (C)**. After seeding, cells rested overnight. B16-F10 cells were treated with a single dose of 250µM DTIC on day 1, GL261-luc2 cells with 20µM temozolomide every other day for 5 days, and CT26 cells with single doses of 10 µg/ml irinotecan, 10 µg/ml oxaliplatin, and 400 ng/ml 5-fluorouracil. After CT, cells of all cell lines were irradiated with either a single dose of 10 Gray (Gy) on day 5, 2 × 5 Gy on days 3 and 5 or 5 × 2 Gy. Cells were analyzed 24 or 48 h after the last treatment.

After CT, cells were irradiated using an X-ray generator (120 kV, 22.7 mA, variable time; GE Inspection Technologies, Germany) with either a single dose of 10 Gray (Gy) on day 5, hypo-fractionated 2 × 5 Gy on days 3 and 5 or norm-fractionated 5 × 2 Gy RT (Figure 1).

### Cell Death Determination and PD-L1 Surface Expression of Tumor Cells

About 24 and 48 h after the last irradiation, tumor cells were harvested for analyses by flow cytometry. For cell death detection and analysis of PD-L1 or PD-L2 surface expression, 0.5–1 × 10^5^ tumor cells were blocked with Fc Block (anti-CD16/32 antibodies, Affymetrix, USA), stained with 7-Aminoactinomycin D (7-AAD Biolegend, USA), AnnexinV-FITC (AxV, Life Technologies and Sigma-Aldrich, USA), and anti-PD-L1-PE-Cyanine7 (Affymetrix, USA) or anti-PD-L2-APC (Biolegend, USA) for 30 min at 4°C in the dark and analyzed using flow cytometry (Gallios, Beckman Coulter, USA). Before use, the anti-PD-L1 (clone MIH5, dilution 125 ng/ml) and anti-PD-L2-APC (clone TY25, 1/100, Biolegend, USA) antibodies were titrated, and the isotype control for every condition was subtracted from the measured PD-L1 or PD-L2 mean fluorescence intensity. Cells negative for AnnexinV-FITC and 7-AAD (AxV^−^/7-AAD^−^) were identified as vital, cells positive for AxV but negative for 7-AAD (AxV^+^/7-AAD^−^) as apoptotic and cells positive for 7-AAD (7-AAD^+^) as necrotic.

### Measurement of Intracellular IFN-Gamma

For intracellular analysis of IFN-gamma, the Cytofix/Cytoperm-Kit (BD Biosciences, USA) protocol was followed. In brief, cells were incubated with the protein transport inhibitor brefeldin A for 4 h at 37°C and 5% CO_2_ to support intracellular cytokine accumulation, and they were then trypsinized and counted. A total of 2 × 10^5^ cells were afterward fixed and permeabilized. Subsequently, cells were stained with an anti-IFN-gamma-PE-Cyanine7 antibody (XMG1.2, BD Biosciences, USA) and analyzed by flow cytometry.

### Measurement of Extracellular IL-6

IL-6 was determined in the supernatants of the tumor cells by ELISA according to the manufacturer’s instructions (IL-6 ELISA kit from BioLegend, USA).

### C57BL/6-B16-F10 Mouse Melanoma Model

C57BL/6 mice (Janvier, Germany) were maintained in a SPF facility under sterile atmosphere at the animal facility of the Friedrich-Alexander-Universität Erlangen-Nürnberg (Franz-Penzoldt-Center). Here, the animals can also be kept after chemoradiation. The animal procedures have been approved by the “Regierung of Mittelfranken” and were conducted in accordance with the guidelines of Federation of European Laboratory Animal Science Associations (FELASA).

About 8- to 10-week-old female C57BL/6 mice were used for the B16-F10 melanoma model: 1 × 10^6^ B16-F10 cells (ATCC, USA) were re-suspended in 200 µl Ringer’s solution and injected subcutaneously into the right flank of the mice on day 0. The tumor volume was monitored using a digital caliper at the given time points and calculated using the following formula: volume (mm^3^) = 0.5 × width^2^ (mm^2^) × length (mm) (28).

The tumor-bearing mice were then randomly assigned to the different treatment groups (group 1: untreated controls, group 2: fractionated RT, and group 3: fractionated RT plus DTIC). Local irradiation of the mice was done as established and published before by our group (28). At day 8, 9, and 10 after tumor induction, local RT with 2 Gy was performed. At day 8 and 10, DTIC (2 mg/mouse) was injected i.p., 2 h after irradiation.

For investigation of PD-L1 expression on B16-F10 tumor cells, the tumors were dissected on day 13 after tumor induction, and single cell suspensions were prepared using a tumor dissociation kit (Miltenyi Biotec, Germany). For separation of dead cells and cell debris, an easycoll-solution based (Biochrom, Germany) density gradient centrifugation was performed. Unspecific binding sites were blocked using anti-CD16/32 (eBioscience, USA) antibodies, and cells were then stained for 30 min at 4°C with fluorescence labeled antibodies: CD45-FITC (eBioScience, USA), PD-L1-PE-Cy7 (eBioscience, USA), and PD-L1-BV421 (BioLegend, USA). Afterward, multicolor flow cytometry was performed using the Gallios Flow Cytometer (Beckman Coulter Inc.).

### Statistical Analyses

The arithmetic mean of replicates, as calculated by flow analysis software Kaluza 1.2 and 1.3 (Beckman Coulter, USA), is depicted. The software Prism 5 (graph pad, USA) was used for statistics. For all analyses, one-tailed Mann–Whitney *U* test was used, unless stated otherwise. Results were considered statistically significant for *p < 0.05, **p < 0.01, and ***p < 0.001.

## Results

### In Particular Fractionated RT Increases PD-L1 Surface Expression on Vital B16-F10 Melanoma Cells

**Figure 2:**
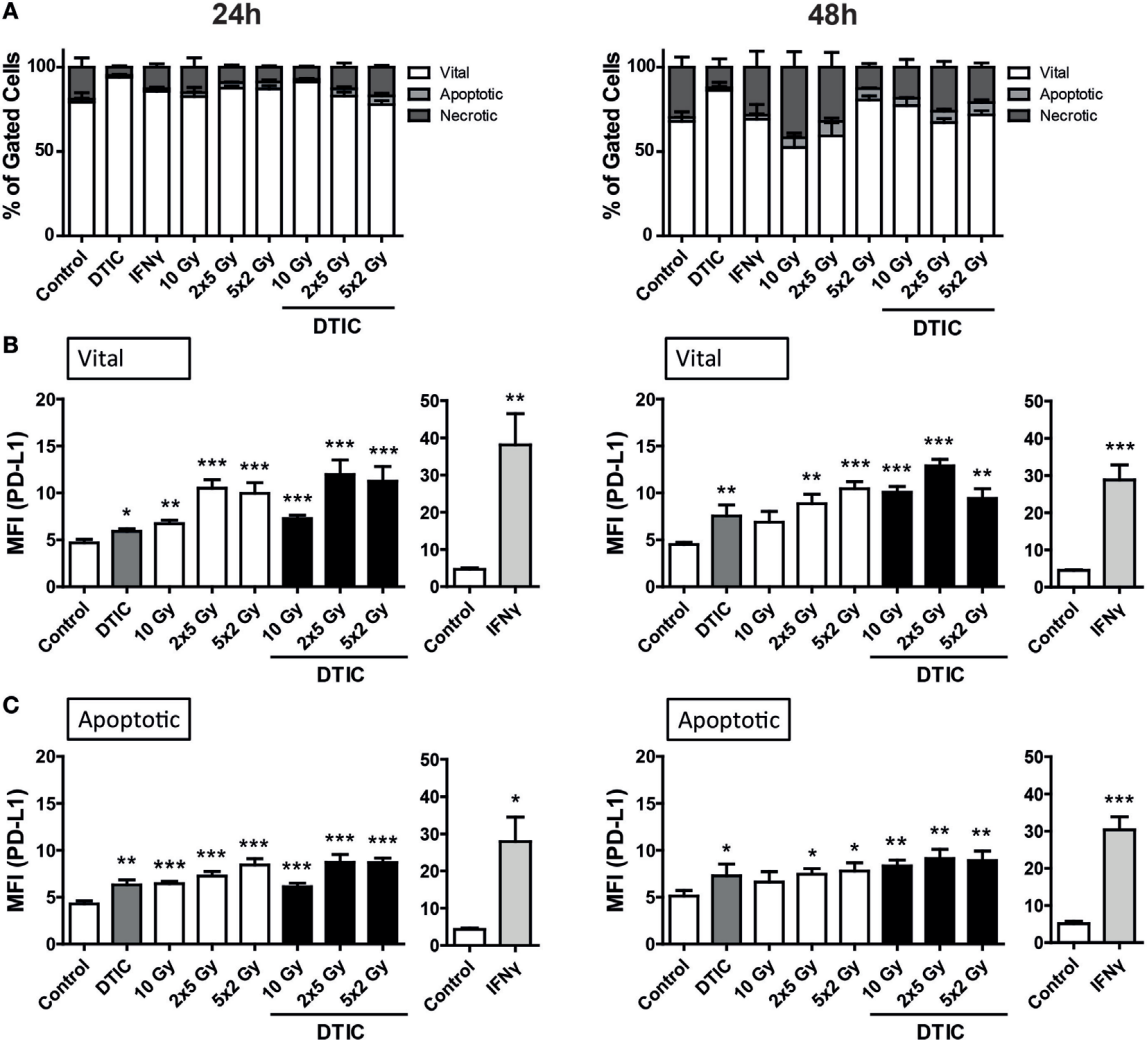
**Cell death and programed cell death receptor ligand 1 (PD-L1) surface expression of B16-F10 melanoma cells after radiation and/or chemotherapy**. The analyses were performed 24 and 48 h after single and multimodal treatments with the chemotherapeutic agent DTIC, differently fractionated radiotherapy, or radiochemotherapy. Cell death was determined by flow cytometry; vital cells (white) are defined as AxV^−^/7-AAD^−^, apoptotic cells (gray) as AxV^−^/7-AAD^+^, and necrotic ones (dark gray) as 7-AAD^+^ **(A)**. PD-L1 surface expression was determined on vital **(B)** and apoptotic **(C)** cells by staining with anti-PD-L1 antibody and consecutive analysis by flow cytometry. DTIC was used at a concentration of 250 µM and recombinant murine interferon-gamma (0.5 ng/ml) served as a positive control **(A–C)**. Joint data of three independent experiments, each performed in triplicates, are presented as mean ± SEM and analyzed by one-tailed Mann–Whitney *U* test as calculated *via* Graph Pad Prism. Each treatment was compared to the control (*p < 0.05; **p < 0.01; ***p < 0.001).

The B16-F10 melanoma cells proved to be highly resistant against radiation, since 24 h after the respective treatments only few tumor cells died *via* apoptosis or necrosis. After 48 h, in particular DTIC plus fractionated RT with 2 × 5 Gy or 5 × 2 Gy induced apoptosis and necrosis, but still over 50% of the melanoma cells were vital (Figure 2A).

To determine whether PD-L1 expression is dependent on the induction of cell death, its surface expression on vital and apoptotic tumor cells (Figures 2B,C) was compared. All tumor cells do express PD-L1 and in particular on vital B16-F10 cells, norm-fractionated and hypo-fractionated RT resulted in the highest increase of surface expression of PD-L1.

Although to a lesser extent but still significant when compared to mock-treated cells, single dose irradiation with 10 Gy or DTIC treatment also led to an increase in PD-L1 surface expression (Figures 2B,C). Combination of DTIC and RT resulted in similar expression levels of PD-L1 compared to only RT-treated cells at an early time point (24 h) after treatment (Figure 2B). Representative histograms of the increased surface expression of PD-L1 of B16-F10 melanoma cells after chemoradiation (RCT) are shown in the Figure S1A in Supplementary Material. Furthermore, a significant increase of PD-L1 expression was also observed on already dying tumor cells after radiation or chemoradiation (Figure 2C).

### In Particular Fractionated RT and TMZ Increase PD-L1 Surface Expression on Vital Glioblastoma GL261-luc2 Cells

**Figure 3:**
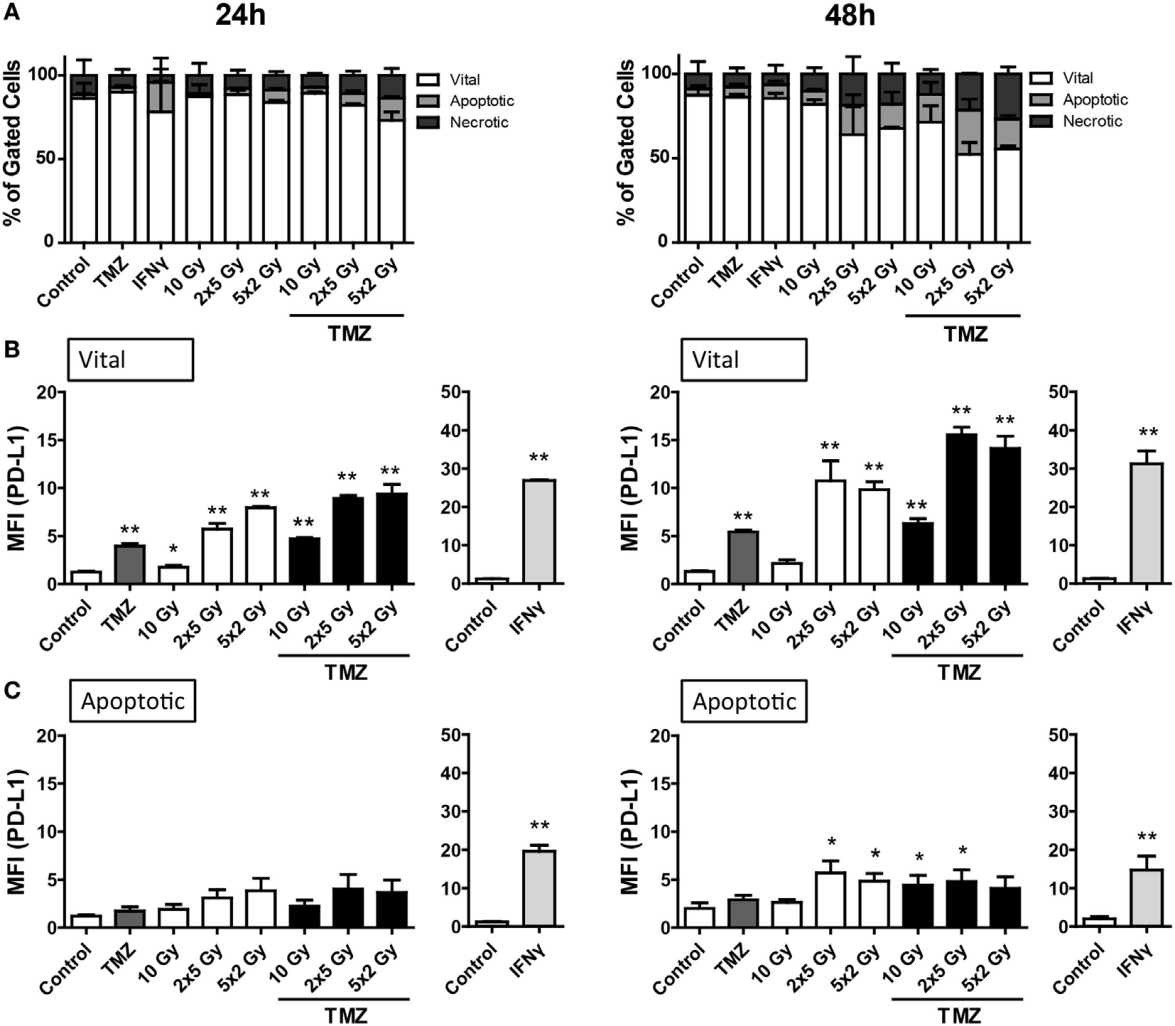
**Cell death and programed cell death receptor ligand 1 (PD-L1) surface expression of GL261-luc2 glioblastoma cells after radiation and/or chemotherapy**. The analyses were performed 24 and 48 h after single and multimodal treatments with the chemotherapeutic agent temozolomide (TMZ), differently fractionated radiotherapy, or radiochemotherapy. Cell death was determined by flow cytometry; vital cells (white) are defined as AxV^−^/7-AAD^−^, apoptotic cells (gray) as AxV^−^/7-AAD^+^, and necrotic ones (dark gray) as 7-AAD^+^ **(A)**. PD-L1 surface expression was determined on vital **(B)** and apoptotic **(C)** cells by staining with anti-PD-L1 antibody and consecutive analysis by flow cytometry. TMZ was used at a concentration of 20µM and recombinant murine interferon-gamma (0.5 ng/ml) served as a positive control **(A–C)**. Joint data of three independent experiments, each performed in triplicates, are presented as mean ± SEM and analyzed by one-tailed Mann–Whitney *U* test as calculated *via* Graph Pad Prism. Each treatment was compared to the control (*p < 0.05; **p < 0.01; ***p < 0.001).

The percentage of apoptotic as well as necrotic murine glioblastoma cells (GL261-luc2) was increased by fractionated RT (2 × 5 Gy and 5 × 2 Gy) or the combination of a single 10 Gy irradiation with TMZ 48 h after the treatments (Figure 3A). Furthermore, a slight, but not significant enhancement of dying or dead cells could be observed when combining TMZ with fractionated RT.

Regarding PD-L1 surface expression, similar to B16-F10 cells, vital tumor cells displayed the highest level, in particular after fractionated RT and/or treatment with TMZ (Figure 3B). Representative histograms of the increased surface expression of PD-L1 of GL261-luc2 cells after chemoradiation (RCT) are shown in the Figure S1B in Supplementary Material. Dying, namely apoptotic, glioblastoma cells displayed a slight, but significant upregulation of PD-L1 expression 48 h after treatment with fractionated RT or chemoradiation (Figure 3C).

### RT and CT Have No Significant Impact on PD-L1 Surface Expression on Colorectal CT26 Tumor Cells

**Figure 4:**
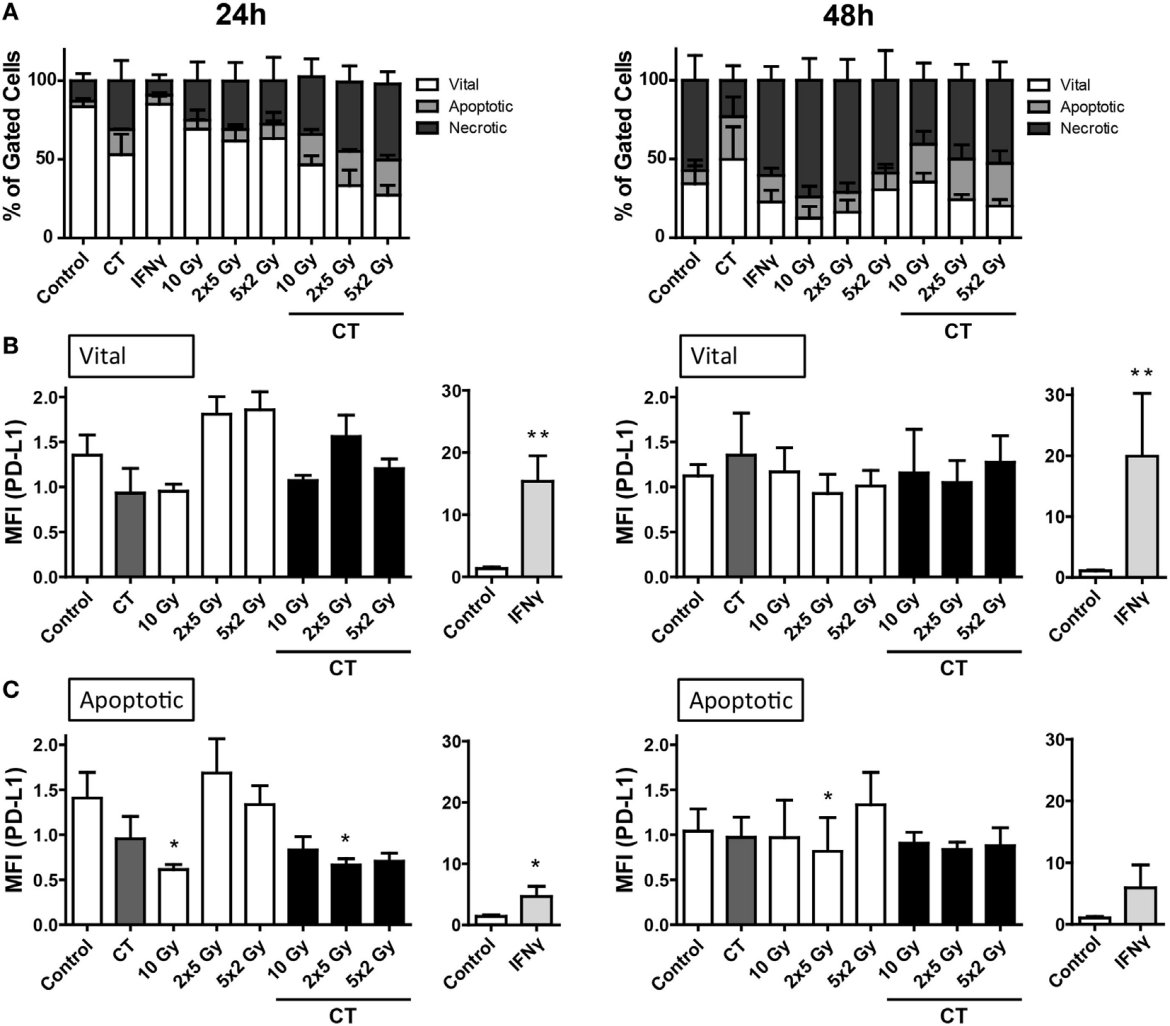
**Cell death and PD-L1 surface expression of CT26 colorectal cancer cells after radiation and/or chemotherapy (CT)**. The analyses were performed 24 and 48 h after single and multimodal treatments with CT consisting of 10 µg/ml irinotecan, 10 µg/ml oxaliplatin, and 400 ng/ml 5-fluorouracil, differently fractionated radiotherapy, or radiochemotherapy. Cell death was determined by flow cytometry; vital cells (white) are defined as AxV^−^/7-AAD^−^, apoptotic cells (gray) as AxV^−^/7-AAD^+^, and necrotic ones (dark gray) as 7-AAD^+^ **(A)**. PD-L1 surface expression was determined on vital **(B)** and apoptotic **(C)** cells by staining with anti-PD-L1 antibody and consecutive analysis by flow cytometry. Recombinant murine interferon-gamma (0.5 ng/ml) served as a positive control **(A–C)**. Joint data of three independent experiments, each performed in triplicates, are presented as mean ± SEM and analyzed by one-tailed Mann–Whitney *U* test as calculated *via* Graph Pad Prism. Each treatment was compared to the control (*p < 0.05; **p < 0.01; ***p < 0.001).

The murine colorectal tumor cells (CT26) were more sensitive to RT and/or CT, and higher percentages of apoptotic and necrotic tumor cells were induced compared to melanoma and glioblastoma cells (Figure 4A). While PD-L1 expression was inducible with recombinant IFN-gamma on the tumor cell surface, neither CT nor the tested RT protocols did significantly increase PD-L1 surface expression on vital and apoptotic colorectal tumor cells, respectively (Figures 4B,C).

### Increased Intracellular IFN-Gamma Expression and Increased Release of IL-6 by Tumor Cells after Fractionated RT and Chemoradiation

The increased surface expression of PD-L1 on melanoma (Figure 2) and glioblastoma cells (Figure 3), particularly after fractionated RT, was independent of contact of the tumor cells with IFN-gamma-producing T cells and may therefore be induced by a tumor cell-dependent mechanism. We therefore analyzed the intracellular IFN-gamma expression as well as the release of IL-6 by B16-F10 and GL261-luc2 cells after norm-fractionated radiation, chemotherapeutic treatment, and chemoradiation.

**Figure 5:**
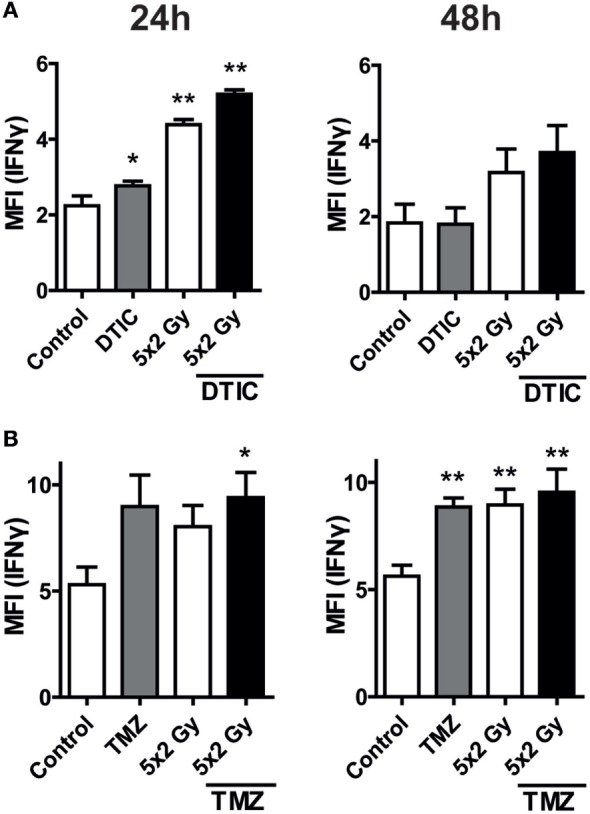
**Intracellular interferon (IFN)-gamma levels after norm-fractionated radiation and/or chemotherapy (CT)**. B16-F10 melanoma **(A)** and GL261-luc2 glioblastoma cells **(B)** were analyzed for intracellular IFN-gamma expression after treatment with either CT with DTIC or temozolomide, 5 × 2 Gy norm-fractionated radiotherapy, or chemoradiation. Data of two independent experiments, each performed in triplicates, are presented as mean ± SEM and analyzed by one-tailed Mann–Whitney *U* test as calculated in Graph Pad Prism. Each treatment was compared to the control (*p < 0.05; **p < 0.01).

An increased expression of IFN-gamma in melanoma cells after in particular radiation and chemoradiation was observed. In glioblastoma cells, IFN-gamma was increased after treatment with TMZ, fractionated RT, and chemoradiation (Figure 5). This parallels with the observed PD-L1 surface expression (Figure 3). Representative histograms of the increased expression of IFN-gamma of B16-F10 and GL261-luc2 cells after radiation and/or CT are displayed in the Figure S2 in Supplementary Material.

**Figure 6:**
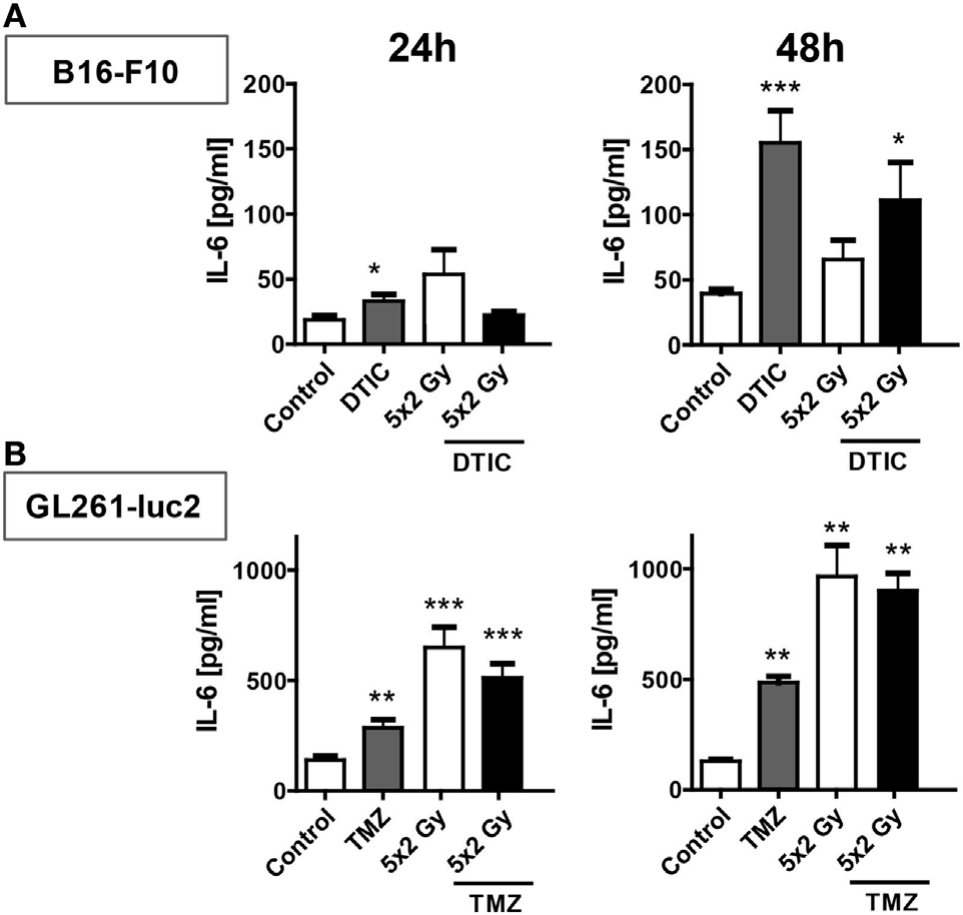
**IL-6 release after norm-fractionated radiation and/or chemotherapy (CT)**. Supernatants of B16-F10 melanoma **(A)** and GL261-luc2 glioblastoma cells **(B)** were analyzed for the concentration of IL-6 by ELISA after treatment with either CT with DTIC or temozolomide, 5 × 2 Gy norm-fractionated radiotherapy, or chemoradiation. Two independent experiments each conducted in technical duplicates were performed. Data are presented as mean ± SEM and analyzed by one-tailed Mann–Whitney *U* test as calculated in Graph Pad Prism. Each treatment was compared to the control (*p < 0.05; **p < 0.01; ***p < 0.001).

In melanoma cells DTIC was the main trigger to induce release of IL-6, in particular 48 h after treatment (Figure 6A). In contrast, in glioblastoma cells norm-fractionated radiation resulted in the highest extracellular concentration of IL-6 (Figure 6B).

### Fractionated RT Plus DTIC Treatment Induces PD-L1 Surface Expression on Melanoma Cells *In Vivo*

**Figure 7:**
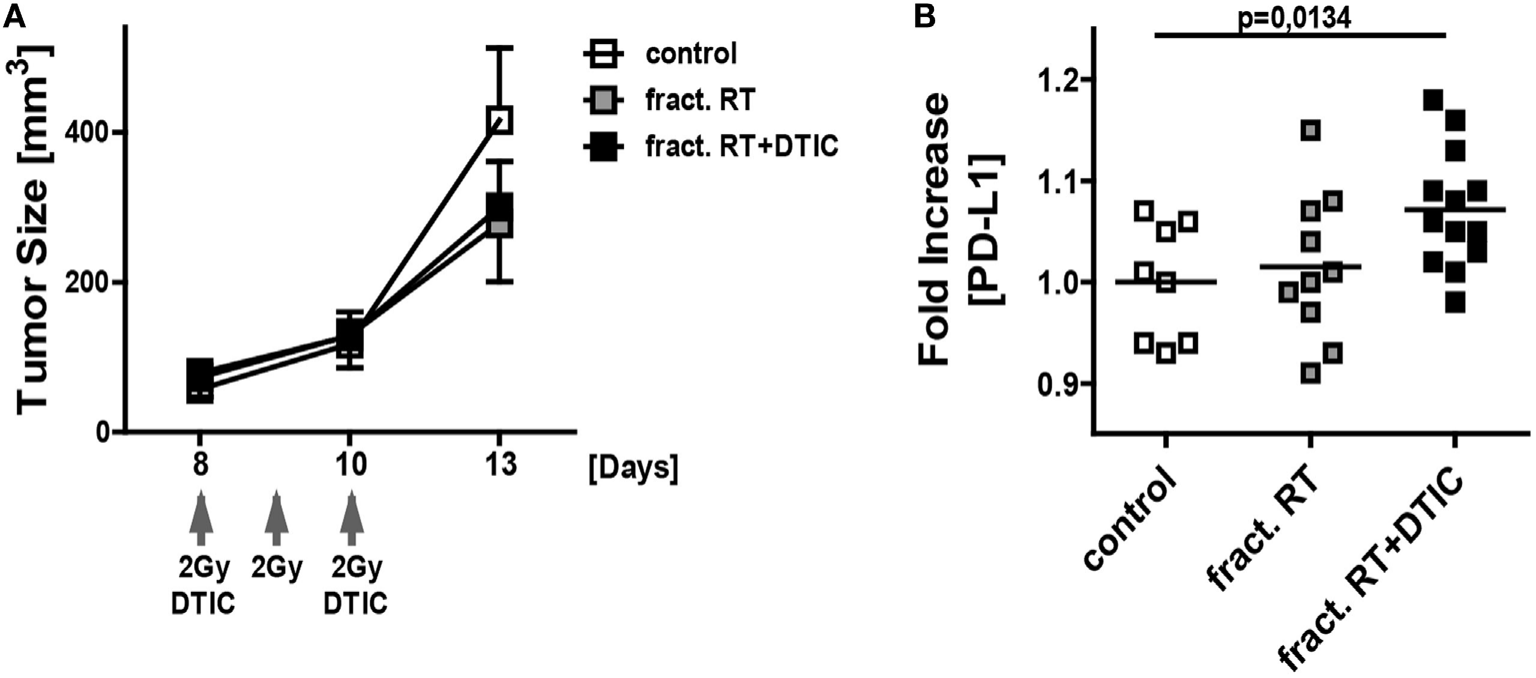
***In vivo* growth and PD-L1 surface expression of B16-F10 tumors after fractionated irradiation and in combination with DTIC treatment**. Growth **(A)** and PD-L1 surface expression **(B)** of B16-F10 tumors in wild-type C57BL/6 mice are displayed. The tumors were initiated on day 0, left untreated or were locally irradiated on day 8, 9, and 10 with the clinically relevant dose of 2 Gray using a linear accelerator. An additional group of mice received DTIC (2 mg/mouse) 2 h after the irradiation at day 8 and 10. For determination of tumor growth **(A)** an electronic caliper was used (*n* ≥ 8 mice/group; data are presented as mean ± SEM). PD-L1 surface expression on the tumor cells (each dot represents the values obtained from an individual tumor of a single mouse; the mean value is displayed as line) **(B)** was analyzed by flow cytometry at day 13. Statistics was analyzed by one-tailed Mann–Whitney *U* test as calculated *via* Graph Pad Prism.

For first clues, whether an upregulation of PD-L1 expression does also occur *in vivo*, the syngenic B16-F10-C57/BL7 ectopic mouse model was chosen (28). For this, B16-F10 tumor-bearing mice were treated with fractionated RT with a clinically relevant dose of 2 Gy or in combination with DTIC administration. Fractionated RT as well as fractionated RT in combination with DTIC did reduce tumor growth on a short term (Figure 7A). To investigate the PD-L1 expression on the melanoma cell surface, single cell suspensions of the tumor cells were prepared and B16-F10 cells were determined as CD45^−^ cells to distinguish them from infiltrating immune cells (CD45^+^ cells). Analyses by flow cytometry revealed that fractionated RT did not lead to an increased PD-L1 expression, but combination of fractionated RT and DTIC resulted in significant increased expression of PD-L1 *in vivo* (Figure 7B).

## Discussion

Several studies have shown a relation between positive response to therapy with immune checkpoint inhibitors and PD-L1 expression (13, 29–31). As such, the PD-1/PD-L1 axis has been regarded as a potential target in tumor tissues. Therefore, identifying whether and how RT or, clinically more relevant, chemoradiation directly results in increased PD-L1 expression is mandatory for optimized multimodal therapies (32). Current data indicate that in particular IFN-gamma, which is secreted by tumor-infiltrating T cells, is responsible for increased expression of PD-L1 on tumor cells (11, 22, 33). The knowledge about direct tumor cell-dependent upregulation of PD-L1 expression upon exposure to RT and/or CT is scarce.

Furthermore, data considering the effect of different fractionation protocols of RT on induction or attenuation of antitumor immune responses are controversial. On the one hand, high single doses were shown to result in improved immunological tumor control compared to hyper-fractionated RT (34, 35). On the other hand, e.g., anti-CTLA-4-mediated immune responses were only observed when combined with fractionated RT (5 × 6 Gy) in murine tumor models (36). We therefore examined the impact of single dose (1 × 10 Gy), hypo-fractionated (2 × 5 Gy), and norm-fractionated (5 × 2 Gy) RT on PD-L1 expression of melanoma, glioblastoma, and colorectal cancer cells. Since in the clinics CT is given in addition to RT, we further focused on single and combined treatment with the respective chemotherapeutic agents. We here show for the first time that CT, namely DTIC and TMZ, significantly enhanced PD-L1 cell surface expression on B16-F10 and GL261-luc2, respectively, albeit the upregulation occurred on a low level (Figures 2 and 3).

Notably, norm-fractionated (5 × 2 Gy) and hypo-fractionated (2 × 5 Gy) irradiation induced the highest PD-L1 expression levels. It has to be stressed that this is primarily an undesired immunosuppressive side effect of RT and therefore should entail addition of IT with anti-PD-L1 antibodies. Of note is that the increased expression of PD-L1 on the tumor cell surface was solely dependent on the tumor cells alone and therefore calls for a tumor cell-dependent effect, since all *in vitro* analyses were carried out in the absence of any immune cells. We revealed an increased expression of intracellular IFN-gamma following in particular chemoradiation in melanoma cells and after TMZ treatment, RT, or RCT in glioblastoma cells, respectively (Figure 5). Just recently, it has been demonstrated that an increased IL-6 expression is in particular observed in PD-L1-expressing human CD68^+^ macrophages compared to PD-L1 low expressing ones (37). We therefore also analyzed IL-6 as possible further intrinsic tumor cell trigger for regulating the expression of PD-L1 after radiation and chemoradiation. The data indicate that in melanoma IL-6 is mainly induced by CT and in glioblastoma by norm-fractionated RT. Detailed pathway analyses on the tumor cell intrinsic triggers for increased expression of immune checkpoints are currently on the way in our lab.

One has to further stress that in preclinical model systems a concerted view is mandatory. While in cell culture RT was sufficient to induce PD-L1 upregulation, the *in vivo* melanoma model showed no significant induction of PD-L1 expression after fractionated RT. However, additional treatment with DTIC enhanced the PD-L1 surface level significantly (Figure 7). Thus, we strongly suggest careful *in vivo* investigations on the matter of different RT schemes and PD-L1 induction to define most beneficial combinations of radioimmunotherapy for the clinics (32).

We further showed that PD-L1 upregulation especially occurs on vital tumor cells and that it was dependent on the tumor entity (Figures 2–4). CT26 colorectal tumor cells did not respond to irradiation with increased PD-L1 expression, even though PD-L1 was inducible upon stimulation with IFN-gamma (Figure 4). This suggests that in distinct tumor entities immune cell-mediated upregulation of PD-L1 expression on tumor cells is predominant, while in others such as melanoma and glioblastoma self-regulatory mechanisms could be dominant. The tumor cell lines used in this study are originated from different tissues, especially with regard to CT26, derived from a mucosal, immunological tissue (38). Here, immune cells might be responsible for upregulation of PD-L1 on the tumor cells. In contrast, glioblastoma is found in a rather immune-privileged area, whereas the skin tissue from which melanoma develops is a relevant immunological barrier. Therefore, tumor cell-dependent mechanisms, independent of immune cells, might be predominant in these cases.

The observed increased PD-L1 expression on apoptotic melanoma and apoptotic glioblastoma cells after chemoradiation additionally calls for combination with agents targeting the PD-1/PD-L1 pathway to overcome the strong immune suppressive effects exerted by apoptotic cells *per se*. They inhibit antitumor immune responses in manifold ways (39).

Moreover, a tumor can only develop by accumulation of many mutations, and it therefore seems reasonable that every tumor entity and every individual tumor will have different mutations that may result in different cell signaling events (27). Especially, melanoma is at high risk of developing mutations due to its exposure to sun-derived UV-light, which has been suggested to play a key role in the susceptibility to anti-PD-1 or anti-PD-L1 treatment (40). Furthermore, among the three examined tumor entities, melanoma is the one with the highest mutational load with a median of 13.2 mutations per Mb, followed by approximately 3.2 in colorectal cancer, and 0.9 in GBM (27). Targeting of PD-1 might be even more efficient than PD-L1, since PD-L2 also binds to PD-1 and in some tumor types PD-L2 expression is more closely linked to IFN-gamma expression and PD-1 signaling than PD-L1 (41). We therefore also checked for the impact of RT, CT, or RCT on increased PD-L2 expression but did not observe it in B16-F10 and GL261-luc2 tumor cells (data not shown).

To summarize, the induction of PD-L1 expression by ionizing irradiation or chemoradiation is dependent on multiple factors such as the individual genetic background, signaling cascades, environment of the tumor, general somatic mutation prevalence, and therefore cannot be generalized. From this, it can be concluded that an anti-PD-L1 therapy concurrent to the classical RT or chemoradiation might not be beneficial in every case, since PD-L1 expression is not the cause of immunosuppression and consecutive tumor cell immune escape in all patients and/or tumor entities. It additionally remains unclear which time point is best for adding immune checkpoint blockade to RCT (4). Our data depict that in dependence on the tumor entity and time after treatment, the surface expression of PD-L1 differs (Figures 2–4) and is partially linked with IFN-gamma expression and IL-6 release (Figures 5 and 6). The key aim of the presented study was to analyze for the first time the impact of in particular chemoradiation on increase of PD-L1 surface expression on tumor cells in the absence of further immune cells. This will presumably give strong hints for the designing of multimodal therapies consisting of RCT with immune checkpoint inhibitors in the future (42).

In a preclinical tumor mouse model, Dovedi et al. suggested an antibody application on the first or last day of RT, but not as late as 7 days after the last treatment (22). Due to the hypothesis that immune cells need some time after therapy to get activated in the periphery and infiltrate into the tumor tissue (43) and the fact that PD-L1 expression needs to be induced in tumor cells first, it can be assumed that application of an anti-PD-L1 treatment can be slightly delayed to classical therapy start within a small timeframe. This assumption and its consequence for CD8^+^ T cell responses needs to be further explored in clinical trials and side effects such as autoimmune reactions will additionally require closely matched monitoring of the treated patients (44).

## Author Contributions

AD performed together with MS most of the practical work, drafted and wrote the manuscript together with UG. MB carried out parts of the practical work and helped to draft the manuscript. MH contributed to the design of the work and the final writing of the manuscript. RF contributed to the design of the work. BF contributed to assay establishments, performed the *in vivo* experiments together with AD, and did analysis and interpretation of the data together with UG and AD. UG drafted and designed the study, drafted the manuscript, and wrote it together with AD and MS.

## Conflict of Interest Statement

The authors declare that the research was conducted in the absence of any commercial or financial relationships that could be construed as a potential conflict of interest.

## Supplementary Material

Figure S1 **Programed cell death receptor ligand 1 (PD-L1) surface expression of B16-F10 melanoma and GL261-luc2 glioblastoma cells after chemoradiation**. The analyses of the increase of PD-L1 surface expression on B16-F10 melanoma **(A)** and GL261-luc2 glioblastoma cells **(B)** were performed 24 h after chemoradiation (RCT) with 10 Gray (Gy), 5 × 2 Gy, or 2 × 5 Gy and DTIC at a concentration of 250 µM. PD-L1 surface expression was determined on vital cells by staining with anti-PD-L1 antibody and consecutive analysis by flow cytometry. Representative histograms of one out of three experiments each performed in triplicates are displayed.

Figure S2 **Interferon (IFN)-gamma expression after norm-fractionated radiation and/or chemotherapy (CT)**. The analyses by flow cytometry of the increase of intracellular IFN-gamma expression in B16-F10 melanoma **(A)** and GL261-luc2 glioblastoma cells **(B)** were performed 24 h after norm-fractionated radiation and/or CT treatment. Representative histograms of one out of two experiments each performed in triplicates are displayed.
