# Supplementary figures and images for: Chemoradiation Increases PD-L1 Expression in Certain Melanoma and Glioblastoma Cells
Source: Front Immunol. 2016 Dec 22;7:610. doi: 10.3389/fimmu.2016.00610 (PMC5177615; doi:10.3389/fimmu.2016.00610)

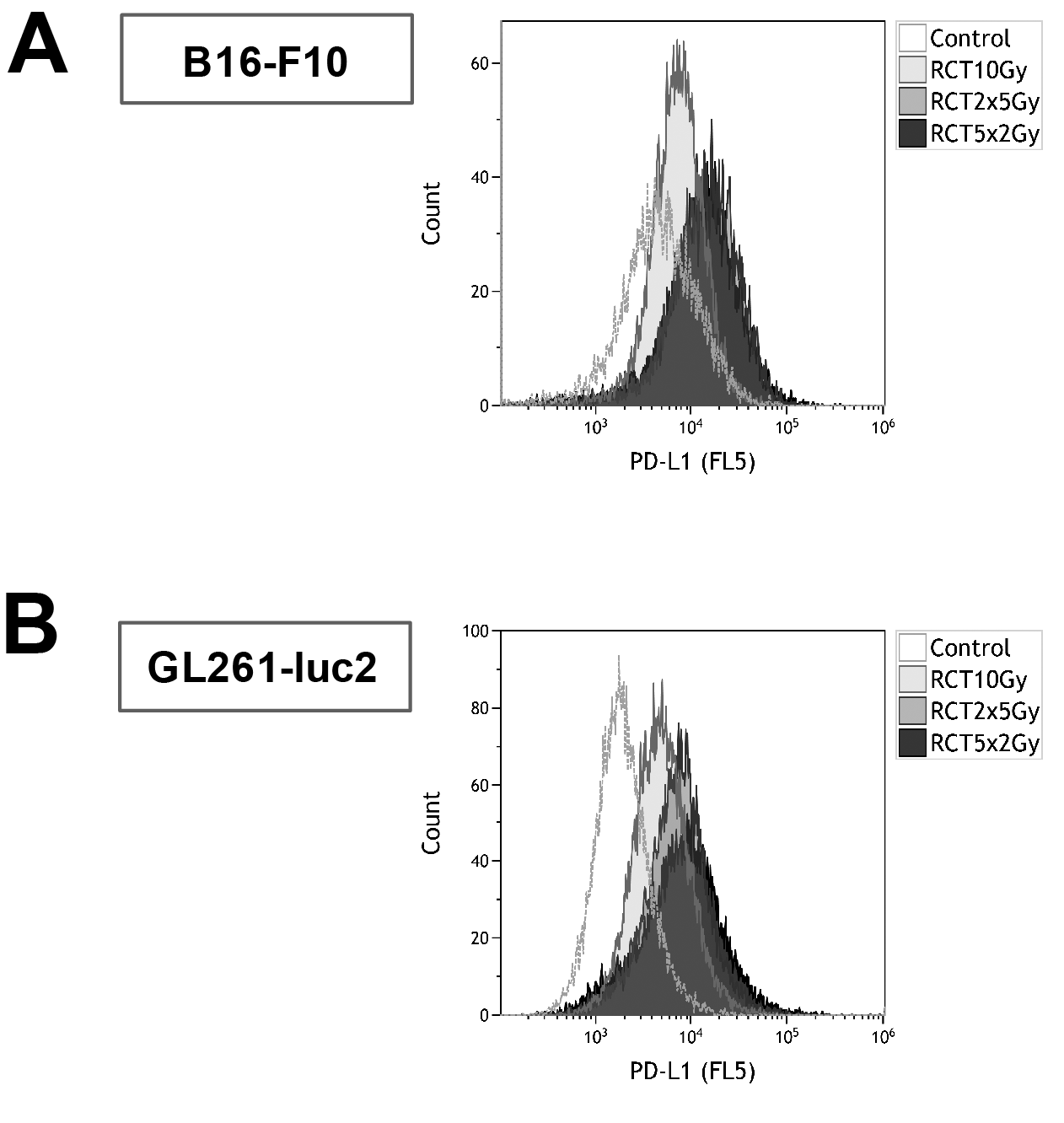

Supplement: Figure S1 — Programed cell death receptor ligand 1 (PD-L1) surface expression of B16-F10 melanoma and GL261-luc2 glioblastoma cells after chemoradiation. The analyses of the increase of PD-L1 surface expression on B16-F10 melanoma (A) and GL261-luc2 glioblastoma cells (B) were performed 24 h after chemoradiation (RCT) with 10 Gray (Gy), 5 × 2 Gy, or 2 × 5 Gy and DTIC at a concentration of 250 µM. PD-L1 surface expression was determined on vital cells by staining with anti-PD-L1 antibody and consecutive analysis by flow cytometry. Representative histograms of one out of three experiments each performed in triplicates are displayed. [file Image_1.tif]

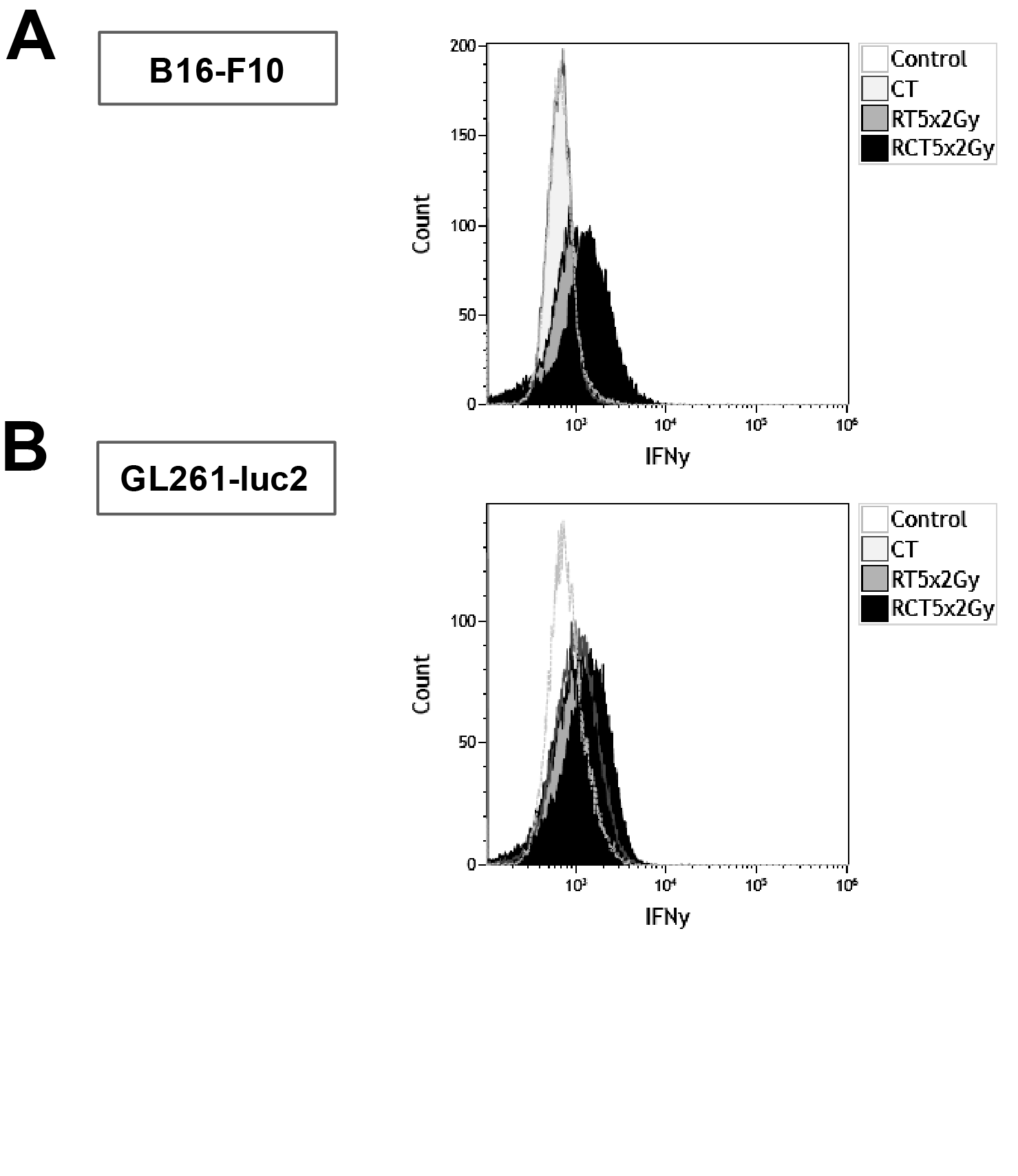

Supplement: Figure S2 — Interferon (IFN)-gamma expression after norm-fractionated radiation and/or chemotherapy (CT). The analyses by flow cytometry of the increase of intracellular IFN-gamma expression in B16-F10 melanoma (A) and GL261-luc2 glioblastoma cells (B) were performed 24 h after norm-fractionated radiation and/or CT treatment. Representative histograms of one out of two experiments each performed in triplicates are displayed. [file Image_2.tif]
